# Investigation of Optimal Afferent Feedback Modality for Inducing Neural Plasticity with A Self-Paced Brain-Computer Interface

**DOI:** 10.3390/s18113761

**Published:** 2018-11-03

**Authors:** Mads Jochumsen, Sylvain Cremoux, Lucien Robinault, Jimmy Lauber, Juan Carlos Arceo, Muhammad Samran Navid, Rasmus Wiberg Nedergaard, Usman Rashid, Heidi Haavik, Imran Khan Niazi

**Affiliations:** 1 SMI, Department of Health Science and Technology, Aalborg University, Aalborg 9220, Denmark; mj@hst.aau.dk; 2 LAMIH, UMR CNRS 8201, Université Polytechnique des Hauts de France, Valenciennes 59313, France; sylvain.cremoux@uphf.fr (S.C.); lucien.robinault@gmail.com (L.R.); jimmy.lauber@uphf.fr (J.L.); juancarlos.Arceo@uphf.fr (J.C.A.); 3 Mech-Sense, Department of Gastroenterology and Hepatology, Aalborg University Hospital, Aalborg 9000, Denmark; m.navid@rn.dk (M.S.N.); rasmus.nedergaard@nzchiro.co.nz (R.W.N.); 4 New Zealand College of Chiropractic, Auckland 1060, New Zealand; heidi.haavik@nzchiro.co.nz; 5 Health and Rehabilitation Research Institute, Auckland University of Technology, Auckland 0627, New Zealand; urashid@aut.ac.nz

**Keywords:** brain-computer interface, neural plasticity, peripheral nerve stimulation, exoskeleton, neurorehabilitation, transcranial magnetic stimulation (TMS)

## Abstract

Brain-computer interfaces (BCIs) can be used to induce neural plasticity in the human nervous system by pairing motor cortical activity with relevant afferent feedback, which can be used in neurorehabilitation. The aim of this study was to identify the optimal type or combination of afferent feedback modalities to increase cortical excitability in a BCI training intervention. In three experimental sessions, 12 healthy participants imagined a dorsiflexion that was decoded by a BCI which activated relevant afferent feedback: (1) electrical nerve stimulation (ES) (peroneal nerve—innervating tibialis anterior), (2) passive movement (PM) of the ankle joint, or (3) combined electrical stimulation and passive movement (Comb). The cortical excitability was assessed with transcranial magnetic stimulation determining motor evoked potentials (MEPs) in tibialis anterior before, immediately after and 30 min after the BCI training. Linear mixed regression models were used to assess the changes in MEPs. The three interventions led to a significant (*p* < 0.05) increase in MEP amplitudes immediately and 30 min after the training. The effect sizes of Comb paradigm were larger than ES and PM, although, these differences were not statistically significant (*p* > 0.05). These results indicate that the timing of movement imagery and afferent feedback is the main determinant of induced cortical plasticity whereas the specific type of feedback has a moderate impact. These findings can be important for the translation of such a BCI protocol to the clinical practice where by combining the BCI with the already available equipment cortical plasticity can be effectively induced. The findings in the current study need to be validated in stroke populations.

## 1. Introduction

Several studies have suggested that brain-computer interfaces (BCIs) can be used for motor rehabilitation after stroke [1,2,3,4,5,6]. Patients are asked to imagine or attempt to perform a movement which is then detected through EEG recordings and translated into a device command that provide feedback to the patient; this can, e.g., be somatosensory afferent or visual feedback. Especially the somatosensory afferent feedback may be important for rehabilitation since it is a motor learning task [7,8]. The underlying physiological mechanism for motor learning is neural plasticity [9], and it has been shown in several studies that the neural plasticity can be induced by BCI training [10,11,12]. In these studies, it has been suggested that Hebbian-associative plasticity is induced by pairing motor cortical activity with relevant somatosensory afferent feedback in the same way as has been done in paired associative stimulation protocols [13]. The difference between BCI and paired associative stimulation is that the motor cortex is activated through imagined movements rather than by stimulation so that brain activation corresponds to the natural activity [14]. The imagined movement must be detected on a single-trial level immediately during the execution to obtain the strict temporal association between motor cortical activity and somatosensory afferent feedback which is needed to induce plasticity [10], as validated in several previous studies [15,16,17,18,19]. It has previously been shown that proprioceptive/somatosensory afferent feedback induces more cortical changes than visual feedback when combined with a BCI [20]. It has been suggested that improved BCI performance could increase the induction of plasticity [11]. Thus, different approaches have been investigated to optimize the BCI performance, such as using different signal processing techniques [21,22,23,24,25,26,27,28], combining two different control signals [29], training the user [30], and facilitate motor imagination [31]. In addition to the technical optimization of the BCI, it is likely that the induction of plasticity can be improved by identifying the optimal type of afferent feedback. Motor-driven orthotic devices and exoskeletons have been used in BCI-related studies and electrical stimulation has been applied with different parameters in terms of stimulation frequency, intensity and location (nerve or muscle stimulation) [3,10,12,32,33,34,35,36,37,38]. These afferent feedback modalities have all been shown to induce plasticity when they were paired with motor cortical activity from imagined or executed movements. Different electrical stimulation frequencies, parameters and locations as well as passive movements have been compared, but due to a large number of combinations it is not feasible to include every combination in a single study. The aim of the current study is to investigate the induction of plasticity when imagined movements are detected in a self-paced BCI system and paired with: (1) single-pulse electrical nerve stimulation, (2) passive movement through a motorized orthotic device, and (3) the combination of electrical nerve stimulation and passive movement. The plasticity induction of the BCI training is evaluated through motor evoked potentials (MEPs) elicited with transcranial magnetic stimulation (TMS). We hypothesize that it is possible to induce plasticity in all three scenarios, but that there is an additive effect by combining electrical stimulation with passive movements.

## 2. Materials and Methods

### 2.1. Participants

Twelve healthy participants were included in the study (four women: 25 ± 4 years). All participants gave their written informed consent prior to the experiment, and they filled in a TMS questionnaire for eligibility based on the recommendations in [39]. All procedures were approved by the Northern B Health and Disability ethical committee (17/NTB/261) New Zealand. All the procedures were carried out according to the Helsinki Declaration.

### 2.2. Experimental Setup

The experiment was divided into three experimental sessions that all followed the same structure where the only difference was the type of afferent feedback: (1) BCI-triggered electrical nerve stimulation, (2) BCI-triggered passive movements, and (3) BCI-triggered electrical nerve stimulation and passive movement. The order of the experimental sessions was randomized, and experimental sessions were separated by at least 24 h. Initially, the participant was seated in a comfortable chair and asked to do 50 self-paced movements, while continuous EEG and EMG was recorded. These movements were used to calibrate the BCI system. After the calibration of the BCI system, the stimulation sites and parameters for the TMS and electrical stimulation were determined. Before the BCI intervention, 15 MEPs were recorded; this was repeated immediately after and 30 min after the BCI intervention. The BCI system was calibrated in each of the three experimental sessions. The BCI intervention lasted until 50 correct parings of imagined movements and afferent feedback were obtained.

### 2.3. Recordings

#### 2.3.1. EEG

Ten channels of continuous EEG were recorded from FP1, F3, Fz, F4, C3, Cz, C4, P3, Pz, and P4 with a sampling frequency of 2048 Hz (Refa amplifiers, TMSi, TMS International, Zuidplas, The Netherlands). FP1 was used to monitor eye movements and eye blinks. The channels were referenced to the right earlobe. During the experiment all channels were below 5 kΩ. The participants were instructed to sit as still as possible and to minimize blinking.

#### 2.3.2. Surface EMG

MEPs were recorded with surface EMG electrodes. Two electrodes (20 mm Blue Sensor Ag-AgCl, AMBU A/S, Ballerup, Denmark) were placed on the belly of the right tibialis anterior muscle in a bipolar configuration with the ground electrode placed on the tibia. The signals were amplified with a custom-made amplifier with a gain of 1000 and a sampling frequency of 4000 Hz. The signals were band-pass filtered from 20–1000 Hz.

### 2.4. Stimulation and Motorized Orthotic Device

#### 2.4.1. Transcranial Magnetic Stimulation

Single pulse TMS was used to elicit MEPs in the tibialis anterior with a Magstim 200 (Magstim Company, Dyfed, UK) using a figure-of-eight double-cone coil with a posterior-anterior current direction. Before recordings, the optimal stimulation site was determined as the site where the largest peak-to-peak amplitude of the MEP in tibialis anterior was elicited compared to the adjacent areas. This area was marked to make sure the coil was placed in the same position for the pre-, post-, and post-30 min intervention measurements. The resting threshold was then determined as the lowest stimulator output where five out of 10 peak-to-peak amplitude MEPs were greater than 50 µV. In the pre-, post-, and post-30 min intervention measurements, 15 stimuli were given at 120% of the resting threshold. Each stimulus was separated by 5–7 s.

#### 2.4.2. Electrical Stimulation

Peripheral nerve stimulation was delivered to the deep branch of the common peroneal nerve supplying the tibialis anterior. The electrical stimulation was delivered through two stimulation electrodes (32 mm, PALS, Platinum, Patented Conductive Neurostimulation Electrodes, Axelgaard Manufacturing Co., Ltd., Fallbrook, CA, USA) that were placed on the skin overlying the nerve with the cathode placed proximal and the anode placed distal. The optimal stimulation site was determined by searching for the location that determined activity in the tibialis anterior without any activity in synergistic or antagonistic muscles (as determined by palpation of the muscles). The motor threshold was then determined as the lowest intensity required to elicit a palpable response in the tibialis anterior tendon. In the two interventions involving electrical stimulation, a single 1-ms wide pulse was delivered with an intensity corresponding to 110% of the motor threshold.

#### 2.4.3. Passive Movements Through the Motorized Orthotic Device

The passive dorsiflexion ankle movement was delivered through a custom-made motorized orthotic device based on the design of an ergometer [40] coupled with a CAHB–21 linear actuator (SKF, Goteborg, Sweden) arranged together to rotate the ankle joint [41,42]. The foot and leg were fixed to the orthosis with straps. The initial position of the orthotic device corresponded to 110° of plantar flexion. The orthosis performed a 15° dorsiflexion rotation around the ankle joint at a constant angular speed of 40°∙s^−1^.

### 2.5. Brain-Computer Interface

**Figure 1:**
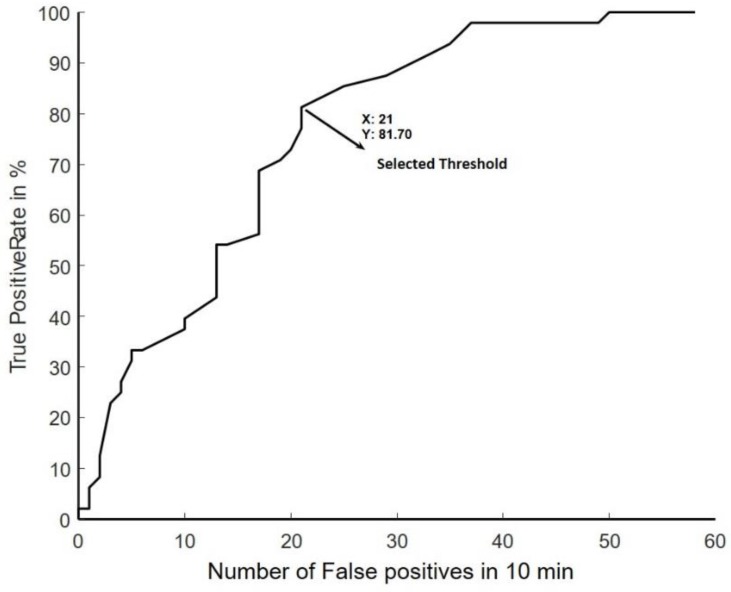
ROC curve, from one training set which was 10 min long to show the trade-off between TPR and FP’s.

The BCI system used in this study has been previously described [11]. Briefly, the system was trained from 50 self-paced movements. From these movements, an EEG signal template of the initial negative phase of the movement-related cortical potentials was extracted from the onset of the movement (determined from the EMG). For this purpose, the EEG was band-pass filtered from 0.05–10 Hz with a 2nd-order zero-phase shift Butterworth filtered, down sampled (at 32 Hz) and filtered with an optimised spatial filter [15] with Cz as the centre electrode. Template matching was used to calibrate the BCI detector as explained in [11,15]. The threshold for detecting the movements was obtained through a receiver operating characteristics curve to achieve a trade-off between the true positive and false positive detections/10 min (length of the training data set) as shown in Figure 1. When the output from the template matching exceeded the threshold, the BCI system registered it as a movement. During the BCI intervention, the BCI system was disabled for five seconds after an event was detected and in this interval the participant indicated if it was a true positive detection or a false positive detection. The participants also indicated the presence of false negatives. Moreover, the detector was disabled if the activity in FP1 exceeded a 125 µV threshold. The BCI system performance was evaluated through the true positive rate (TPR) and number of false positive detections per minute (FPm). 

### 2.6. Statistical Analysis

In the statistical analysis, we were concerned with three questions: (a) Did the BCI performance have an effect on the MEP amplitude? (b) did the pre- to post- and post-30 treatment effect of electrical stimulation (ES), passive movements (PM), and electrical stimulation combined with passive movements (Comb) induced plasticity? (c) What was the difference in these effects across the three paradigms? We evaluated (a) in terms of absolute units (mV) using blinded covariate analysis [43], and (b), (c) in terms of both absolute units and relative units (% change). For absolute units, we computed the peak-peak amplitudes of the MEPs and averaged them across the 15 trials. For the relative units, we computed subject wise % changes from the averaged peak-peak amplitudes of the MEPs as (post- − pre-)/pre- × 100.

To answer these questions, we setup linear mixed regression models. In all the models, time, session, and subject were codified as categorical variables. All the remaining variables were codified as continuous. The statistical analysis was performed in R (R Foundation for Statistical Computing) version 3.5.0. lme4 package version 1.1-17 was used for fitting all the models [44].

For question (a), four performance metrices were considered as covariates: (i) true positive rate (TPR), (ii) false positives per minutes (FP_m_), (iii) time taken to complete the task (T_t_), and (iv) total number of movement repetitions executed (M_r_). Following linear mixed model, presented in R formula syntax, was used for this purpose:

(1) $${\mathrm{MEP}}_{\mathrm{abs}}\;\sim \;1+{\mathrm{MEP}}_{\mathrm{pre}}+\mathrm{Time}+\mathrm{TPR}+{\mathrm{FP}}_{m}+T_{t}+M_{r}+(1|\mathrm{Subject})$$

This model estimates MEP amplitudes at two time points (post- and post-30) while also considering the effect of pre- MEP amplitudes (MEP_pre_), TPR, FP_m_, Tt, and Mr. The random intercept term for subjects (1|Subject) entered into the model, estimates the variance across the subjects. This model was fitted to data from the three sessions. However, the Session variable was not added to the model to keep the analysis blinded as suggested by Kunz et. al. [43]. This procedure has two benefits. First, it allows for an unbiased selection of covariates as the selection is done before obtaining the final results. Second, it leaves out unnecessary covariates which can potentially act as noise in the final model, thus, improving the statistical power. It was planned a priori that a covariate which explained greater than or equal to 5% of the variance in the data will be considered as potentially having a significant effect on the MEP amplitudes. Furthermore, it will be added to models used for answering questions (b) and (c) in order to statistically control for its effect. Semi-partial R^2^ statistic was obtained using the Kenward-Roger method as a measure of explained variance. The r2glmm package version 0.1.2 was used for this purpose [45].

For questions (b) and (c), following model, expressed as an R formula, was used for absolute units:

(2) $${\mathrm{MEP}}_{\mathrm{abs}}\;\sim \;1+\mathrm{Session}\times \mathrm{Time}+{\mathrm{MEP}}_{\mathrm{pre}}+T_{t}+\left(1│\mathrm{Subject}:\mathrm{Session}\right)$$

This model estimates MEP amplitude across the three sessions (ES, PM, Comb) at both time points (post-, post-30) while adjusting for the pre- MEP amplitudes. This model also controls for total time taken to complete the task as it explained more than 5% of the variance in MEP amplitudes. This model is similar to model 1 suggested by Twisk et al. [46] with two important improvement. First, as the subjects across the sessions were same and it is reasonable to assume that the subjects respond differently to the three paradigms, thus, we used a subject and session wise random intercept (1│Subject:Session) to estimate the between subject variance. This model also suits to the repeated measures design of this study. Second, as the MEP amplitudes are always positive and are not normally distributed, we used Gamma distribution to model the data. The choice of the link function (identity or log) was evaluated using Akaike information criterion corrected for small samples (AICc). The AICc penalises both under fitting and over fitting. We used the log link.

For relative units, we setup the same model with the exception that we used Gaussian distribution and identity link. The residuals of the model were normally distributed. The model, expressed as an R formula, is given below:

(3) $${\mathrm{MEP}}_{\%}\;\sim \;1+\mathrm{Session}\times \mathrm{Time}+{\mathrm{MEP}}_{\mathrm{pre}}+T_{t}+\;\left(1│\mathrm{Subject}:\mathrm{Session}\right)$$

Significance level was set at 0.05. Effects estimated by the model were reported with their standard errors. Pair-wise contrasts were performed with Tukey’s HSD method.

## 3. Results

### 3.1. BCI Performance

The performance metrices and the corresponding variance explained statistics are given in Table 1. Time taken to complete the task explained 6% of the variance in MEP amplitudes and, therefore, was included as a covariate to statistically control its effect in the subsequent models used to estimate MEP_abs_ and MEP_%_.

The linear trends between T_t_ and MEP_abs_ and MEP_%_ estimated by the statistical models are given in Table 2. These results suggest that the time taken to complete the task did not have a statistically significant effect on the MEP amplitudes.

**Figure 2:**
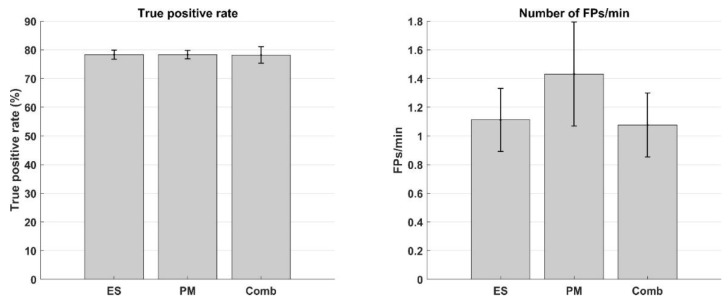
(**Left**) True positive rate (mean ± standard error) across all subjects. (**Right**) Number of false positive detections per minute (mean ± standard error) across all subjects. ‘ES’: Electrical stimulation, ‘PM’: Passive movement, and ‘Comb’: Combined electrical stimulation and passive movement.

As the remaining performance metrices did not explain considerable (R^2^_partial_ < 5 %) variance in the data, they were not added to the statistical models and, thus, their trends were not estimated. To further elaborate on the differences in TPR and FP_m_ across sessions, their means and standard errors are given in Figure 2. One-way ANOVAs suggested that there was no difference between the sessions in terms of both the true positive rate (F(2, 22) = 0.001; *p* = 1.0) or number of false positive detections per minute (F(2, 22) = 0.59; *p* = 0.57). These results together with the explained variance statistic suggest that none of the BCI performance metrices had any significant effect on the MEP amplitudes.

### 3.2. MEP Size

**Figure 3:**
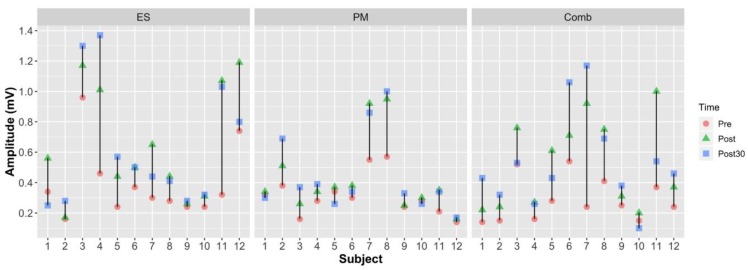
Peak-peak MEP amplitudes for all the subjects for pre- to post- and post-30 raw values.

The peak-peak MEP amplitudes for the subjects are plotted in Figure 3. The individual trends suggest that there was a larger increase in pre- to post-MEP amplitude in case of ES and Comb compared to PM.

The pre- to post-effect sizes estimated from the statistical models are given in Table 3. These effects were computed with T_t_ set to its mean value (13.42 min). Similarly, pair-wise contrasts across the sessions at the two time points are given in Table 3. The pair-wise contrasts across sessions and time are given in Table 4 and Table 5, respectively. As a log link was used in modelling the MEP amplitudes in absolute units, thus the contrasts performed on the log scale are presented as ratios on the response scale. 

These results suggest that the three paradigms increased (*p* < 0.05) the MEP amplitudes from pre- to post- and post-30 in terms of the absolute units. The effect sizes of Comb paradigm were larger than ES and PM, although, these differences were not statistically significant (*p* > 0.05). 

## 4. Discussion

The study confirmed that neural plasticity can be induced by BCI-triggered electrical stimulation or passive movements. However, the three afferent feedback modalities did not differ in their effects on plasticity. The contrasts suggested that electrical stimulation consistently resulted in slightly higher mean MEP amplitudes than passive movement and the combination of passive movement with electrical stimulation consistently resulted in slightly higher mean MEP amplitudes than electrical stimulation alone. 

### 4.1. Effect of BCI-Triggered Afferent Feedback

All three types of afferent feedback induced neural plasticity when combined with the BCI, but there was no statistically significant difference between the types of afferent feedback although larger percentage changes from pre-to post- and pre-to post-30 were obtained when electrical stimulation was involved. However, there was a large variability between participants, presumably due to factors such as attention and time of the day [47]. It is also likely that there might be a difference in the amount and type of sensory feedback that is sent to the brain, which depends on the activation of afferents and the type of receptors that are active during the electrical stimulation (no movement of the foot) and the passive movement. For the electrical stimulation, only the nerve innervating the tibialis anterior was stimulated, thus only low-threshold afferents were recruited [48]. Conversely, during the passive movements, cutaneous and high threshold afferents would be activated because of joint movement. Moreover, other non-muscle afferents should also be active via a transcortical loop resulting in an activation which was close to a voluntary dorsiflexion [48,49]. However, different types of sensory feedback may have different effects on the cortical motor networks when applied as part of an intervention [50]. The latency of the different types of afferent feedback to reach cortex was presumably similar [51,52]. Moreover, another advantage of having the electrical stimulation over peripheral nerve trunk is that it generates contraction facilitated via central pathways by maximizing the afferent (sensory) volley to spinal cord where sensorimotor integration occurs, resulting in optimal recruitment of the spinal motor neuron [28]. It is known in the literature that, If the central contribution is maximized it can lead to reduced muscle atrophy which in turn can be used to restore movement in persons with movement disorders [53].

The observation of no difference between electrical stimulation and passive movements supports previous findings by Mrachacz-Kersting et al. [34]. This means that the choice of the type of afferent feedback modality may not be crucial, and it should rather be selected based on the available equipment in the rehabilitation centers or by the comfort of the patient. However, contrary to the previous study by Mrachacz-Kersting et al. [34], in the current study the BCI operated in a self-paced way. 

The pros of the electrical stimulation are that it is low-cost, and it has been used by many, but the cons are that electrical stimulation can lead to muscle fatigue (especially when using functional electrical stimulation) and it may be painful for some patients (depending on the stimulation parameters). The pros of the motor-driven orthotic device are that there is no muscle fatigue and the movement can be executed with great precision, so it mimics a natural movement. The cons of the motor-driven orthotic device are that it is more expensive that the electrical stimulation and it is less portable. To overcome this latter disadvantage, we developed and used a transportable motorized ergometer [34,35]. It remains to optimize the passive-movement feedback so that it is as close as possible to the dynamic of a real movement. 

### 4.2. Neural Mechanisms

It has been suggested in several BCI studies [4,10,11,12] that the neural mechanisms for the changes observed in BCI intervention studies are similar to that of paired associated stimulation [13], which rely on long-term potentiation properties such as rapid onset, persistence on cessation of stimulation and associativity but with likely differences in the origins of those effects [54]. The increase in cortical excitability was retained at least for 30 min following the intervention [55]. It has previously been shown that this this type of BCI intervention is specific [4,10], which is another indication of that it is long-term potentiation-like plasticity that is induced. Furthermore, the origin of the neural plastic changes were not assessed, but it has been suggested that it is likely to be changes in the cortical plasticity [10,11,12,34].

### 4.3. Limitations

In the current study, we did not detect a statistically significant difference for the alternative hypothesis that ES and PM combined (Comb) have a larger effect in terms of inducing cortical excitability. As the estimated effect sizes supported this hypothesis, the potential reason for not achieving statistical significance seems to be the small sample size (n = 12). Sample size for this study was based on the previous studies [10,11,12,34,55] in which it ranged between 8 and 12. Future studies can plan a better sample size by using the estimated effects and their standard errors reported in the current study. Another limitation is the effect of the BCI intervention without sensory feedback was not tested as well as afferent feedback without the BCI intervention. However, it has previously been shown that 50 electrical stimuli or passive movements alone do not change the size of the MEPs [10,12]. The same has been found for the BCI intervention without any afferent feedback [10,12]. All findings in the current study are based on healthy volunteers which limit the generalization/transfer of the results to stroke rehabilitation. However, similar work has been done for the upper limb where stroke patients have received upper limb BCI training using functional electrical stimulation [56] and rehabilitation robots where functional improvements have been shown [3,5,57]. It is likely that similar beneficial effects may be observed for the lower limbs as well [4].

## 5. Conclusions

It was shown that neural plasticity could be induced with electrical stimulation, passive movement and the combination of electrical stimulation and passive movement when combined with a BCI. 

In conclusion, current study indicates that timing of movement imagery and afferent feedback is the main determinant of inducing cortical plasticity whereas the specific type of feedback has moderate impact. These different types of feedback can be used based on the most practical and convenient one for the patient and caregivers should be selected. This, or a similar, investigation need to be performed with stroke patients in future studies to investigate if the results from healthy subjects can be translated to stroke patients. In such studies, other important aspects can also be included, such as an extended training period, and the measurement of clinically relevant outcomes and their correlation with induction of cortical excitability. Moreover, the usability of such a BCI combined with either electrical stimulation or passive movement need to be investigated. These investigations can provide insights into the transfer of BCI enabled interventions to clinical practice or potential use at home.

## Figures and Tables

**Table 1:** BCI performance metrices and percentage of variance explained in MEP amplitudes by each metric.

Performance Metric	Mean [min, max]	Variance Explained Partial R^2^ (%)
TPR	78.13 [58.82, 96.15]%	1.5
FP_m_	1.21 [0.22, 4.62]	0.8
T_t_	13.42 [7, 23] min	6.0
M_r_	64.36 [52, 85]	0.3

**Table 2:** The linear trends between T_t_ and MEP_abs_ and MEP_%_ estimated by the statistical models.

Trend for T_t_	Estimate	Std. Error	***p*, H_0_:μ = 0**
With MEP_abs_ (mV/min)	0.01	0.02	*z* = 0.65, *p* = 0.51
With MEP_%_ (%/min)	1.32	2.79	t[31] = 0.47, *p* = 0.63

**Table 3:** Pre- to post- and post 30-effect sizes along with the standard errors estimated from the statistical models.

**Session**	**Time**	**MEP_abs_ (mV)**	**Std. Error (mV)**	***z*, *p*, H_0_:μ = 0**
ES	post-	0.21	0.05	*z* = −6.97, *p* < 0.001
PM		0.17	0.03	*z* = −8.83, *p* < 0.001
Comb.		0.22	0.04	*z* = −7.98, *p* < 0.001
ES	post-30	0.20	0.05	*z* = −7.13, *p* < 0.001
PM		0.19	0.04	*z* = −8.40, *p* < 0.001
Comb.		0.22	0.04	*z* = −8.00, *p* < 0.001
**Session**	**Time**	**MEP_%_ (%)**	**Std. Error (%)**	**t[df], *p*, H_0_:μ = 0**
ES	post-	81.26	32.86	t[35.29] = 2.47, *p* = 0.02
PM		41.16	29.59	t[36.37] = 1.39, *p* = 0.17
Comb.		94.90	27.99	t[37.05] = 3.39, *p* < 0.01
ES	post-30	80.44	32.86	t[35.29] = 2.45, *p* = 0.02
PM		56.37	29.59	t[36.37] = 1.91, *p* = 0.06
Comb.		104.69	27.99	t[37.05] = 3.74, *p* < 0.001

**Table 4:** Contrasts across sessions and their standard errors estimated from the statistical models.

**Contrast**	**Time**	**Ratio**	**Std. Error (Ratio)**	***z*, *p*, H_0_:μ = 1**
ES/PM	post-	1.23	0.25	*z* = 0.99, *p* = 0.57
ES/Comb.		0.94	0.19	*z* = −0.32, *p* = 0.95
PM/Comb.		0.77	0.15	*z* = −1.32, *p* = 0.39
ES/PM	post-30	1.08	0.22	*z* = 0.39, *p* = 0.92
ES/Comb.		0.91	0.18	*z* = −0.46, *p* = 0.89
PM/Comb.		0.84	0.17	*z* = −0.85, *p* = 0.67
**Contrast**	**Time**	**Difference (%)**	**Std. Error (%)**	**t[df], *p*, H_0_:μ = 0**
ES − PM	post-	40.10	29.90	t [42.22] = 1.34, *p* = 0.38
ES − Comb.		−13.64	301.16	t [42.00] = −0.45, *p* = 0.89
PM − Comb.		−53.74	29.77	t [42.33] = −1.81, *p* = 0.18
ES − PM	post-30	24.06	29.90	t [42.22] = 0.81 *p* = 0.70
ES − Comb.		−24.25	30.16	t [42.00] = −0.80, *p* = 0.70
PM − Comb.		−48.31	29.77	t [42.33] = −1.62, *p* = 0.25

**Table 5:** Contrasts across time and their standard errors estimated from the statistical models.

**Contrast**	**Session**	**Ratio**	**Std. Error (Ratio)**	***z*, *p*, H_0_:μ = 1**
post-/post-30	ES	1.03	0.08	*z* = 0.42, *p* = 0.67
	PM	0.94	0.07	*z* = −1.13, *p* = 0.26
	ES+PM	1.00	0.08	*z* = 0.05, *p* = 0.96
**Contrast**	**Session**	**Difference (%)**	**Std. Error (%)**	**t[df], *p*, H_0_:μ = 0**
post- − post-30	ES	0.82	16.74	t [33] = 0.05, *p* = 0.96
	PM	−15.22	16.74	t [33] = −0.91, *p* = 0.37
	ES+PM	−9.79	16.74	t [33] = −0.59, *p* = 0.56
